# P-1139. Leveraging Instant Orders to Create a Semi-Automated Process for Candida auris Ring Surveillance

**DOI:** 10.1093/ofid/ofaf695.1333

**Published:** 2026-01-11

**Authors:** Alexander S Plattner, Carole Leone, Megan Dethloff, Josephine Fox, Lydia J Grimes- Jenkins, Carlee Hoxworth, Ashley Lloyd, Kevin M Heard, Sandra McCormick, Nicholas B Hampton, Ronald Jackups, Kevin O’Bryan, Patrick J Reich

**Affiliations:** WashU Medicine / St. Louis Children's Hospital, St Louis, MO; BJC Healthcare, Saint Louis, Missouri; BJC Health System, St. Louis, Missouri; Barnes-Jewish Hospital, Saint Louis, Missouri; Barnes-Jewish Hospital, Saint Louis, Missouri; BJC HealthCare, St Louis, Missouri; St. Louis Children's Hospital, Saint Louis, Missouri; BJC HealthCare, St Louis, Missouri; BJC HealthCare, St Louis, Missouri; BJC Healthcare, Saint Louis, Missouri; Washington University in St Louis, St Louis, Missouri; Washington University in St Louis, St Louis, Missouri; Washington University School of Medicine, Saint Louis, Missouri

## Abstract

**Background:**

*Candida auris* is a growing threat among healthcare-associated infections due to its resistance to several antifungal classes and potential for spread within healthcare settings. Ring surveillance involves polymerase chain reaction (PCR) testing from axilla and inguinal fold swabs performed weekly on patients hospitalized near known *C. auris* cases. In our healthcare system, over 200 manual *C. auris* ring surveillance orders were entered weekly by infection preventionists (IPs); a burdensome process, preventing other tasks and contributing to burnout.

**Figure:**
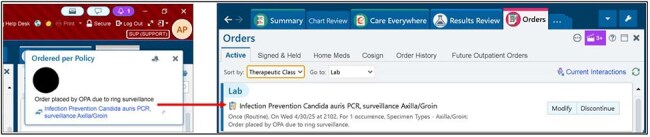
Instant Orders Demo Example of Instant Order notification when activated, followed by placement of surveillance PCR as an active order. The clipboard and lightning icon indicates that this order was placed by Instant Order functionality.

**Figure:**
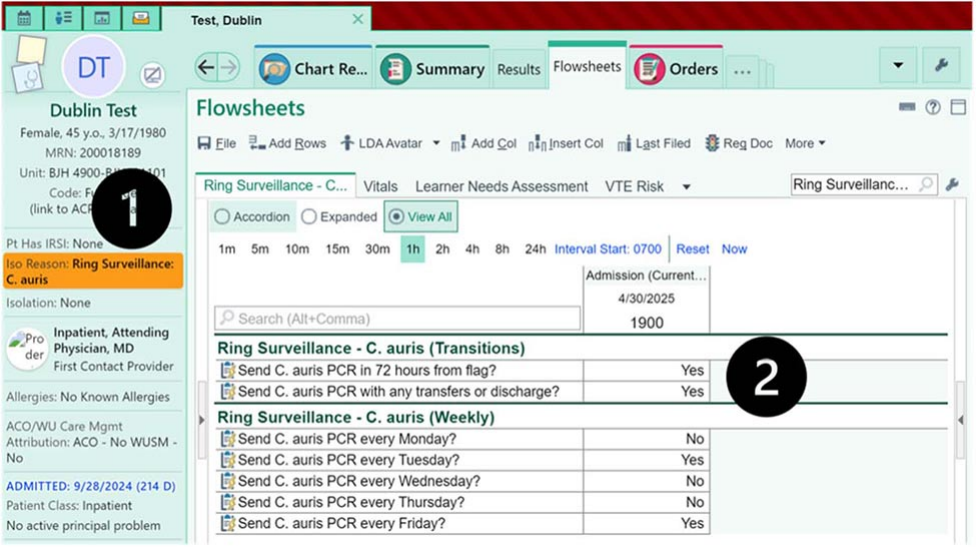
C auris Ring Surveillance Flag and Instant Orders Flowsheet Screenshot of a test patient in Epic showing active C. auris ring surveillance flag (1) and completion of ring surveillance flowsheet (2). Both an active flag and an affirmative response (i.e. “yes”) in desired flowsheet row(s) are required for instant order placement.

**Methods:**

IPs were surveyed to estimate work hours spent manually ordering *C. auris* PCRs. A semi-automated ring surveillance ordering process was implemented using Epic’s “Instant Orders” and non-interruptive OurPractice Advisories (OPAs) to place *C. auris* PCR orders (Fig 1). The process covered three scenarios: 1) after ring surveillance flag placement, 2) upon transfer or discharge order signing, and 3) weekly ordering. An active ring surveillance flag and IP completion of a flowsheet were required for each individual OPA to fire (Fig 2). OPAs were piloted for 10 days on three units before healthcare system-wide implementation. Epic reports were used to track OPA performance.

**Figure:**
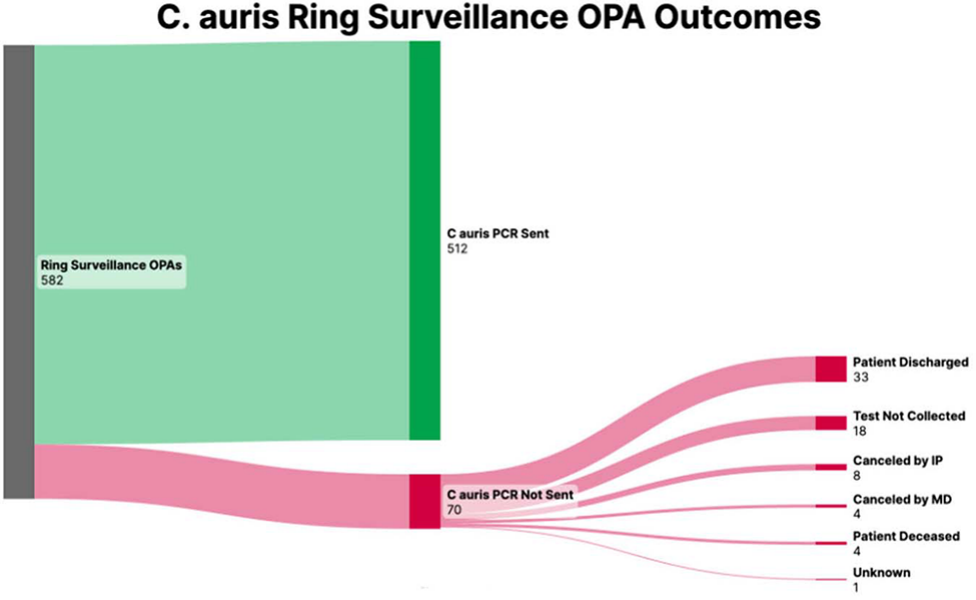
Sankey Diagram of OPA Outcomes Sankey diagram showing outcomes of all OPA firings from the first 28 days. For OPAs that did not lead to performance of surveillance PCR, reasons for lack of testing are provided.

**Figure:**
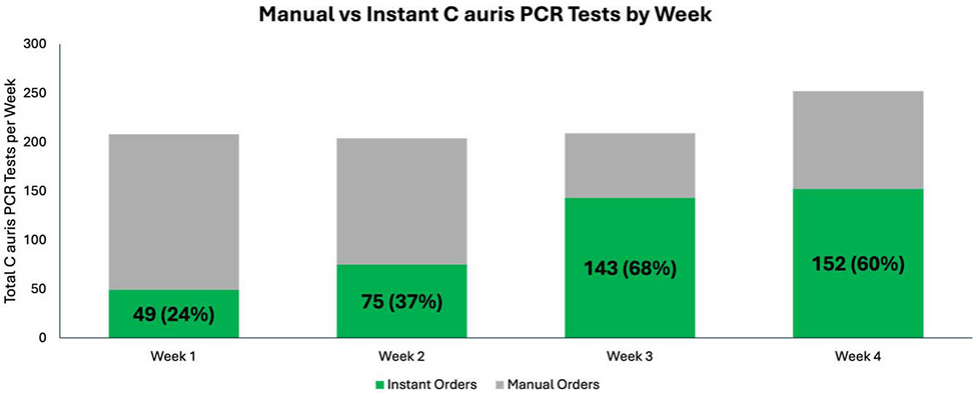
Manual vs Instant C auris PCR Tests by Week Weekly C auris testing following implementation, comparing instant vs manual orders for each week. The total number and percent of total orders placed by OPAs for each week is indicated.

**Results:**

IP surveys indicated 54.5 hours per week spent on tasks related to C. auris PCR ordering prior to implementation. In the first 28 days following implementation, 582 *C. auris* PCR orders were placed by OPAs, resulting in 512 (88%) tests performed (Figure 3). Only 8 (1.4%) of orders were canceled by IPs, suggesting a low number of unexpected firings. The majority of OPAs without a subsequent test were due to patient discharge (33; 47%) or lack of team collection (18; 26%). PCR collection prior to discharge increased from 8.3% in the two weeks prior to the alert to 61% following the alert. A weekly increase in instant orders was observed as IPs became more familiar with the process (Fig 4).

**Conclusion:**

A semi-automated process for *C. auris* surveillance was successfully implemented with minimal unintended orders and higher discharge PCR collection rates. The semi-automated process saves substantial time for IPs based on pre-implementation IP surveys. This approach enhances infection control and is replicable in other healthcare settings.

**Disclosures:**

Ronald Jackups, Jr., MD, PhD, Siemens: Contracted research|Werfen: Advisor/Consultant|Werfen: Contracted research

